# A Functional Polymorphism-Mediated Disruption of EGR1/ADAM10 Pathway Confers the Risk of Sepsis Progression

**DOI:** 10.1128/mBio.01663-19

**Published:** 2019-08-06

**Authors:** Feng Chen, Yan Wang, Wenying Zhang, Yujie Cai, Tian Zhao, Hui Mai, Shoubao Tao, Wenyan Wei, Jia Li, Xiongjin Chen, Xiaohui Li, Pei Tang, Weihao Fan, Jingqi Yang, Mingqian Ou, Furong Lu, Zhipeng Lai, Huiyi Chen, Ting Zou, Furong Sun, Yiming Shao, Lili Cui

**Affiliations:** a Institute of Neurology, Guangdong Key Laboratory of Age-Related Cardiac and Cerebral Diseases, Affiliated Hospital of Guangdong Medical University, Zhanjiang, China; b Key Laboratory of Biomedical Information Engineering of Ministry of Education, School of Life Science and Technology, Xi’an Jiaotong University, Xi’an, China; c The Intensive Care Unit, Guangdong Key Laboratory of Age-Related Cardiac and Cerebral Diseases, Affiliated Hospital of Guangdong Medical University, Zhanjiang, China; d Department of Cardiology, Affiliated Hospital of Guangdong Medical University, Zhanjiang, China; e The Central Hospital of Wuhan, Tongji Medical College, Huazhong University of Science and Technology, Wuhan, China; f Department of Health Technology and Informatics, Hong Kong Polytechnic University, Kowloon, Hong Kong, China; University of Minnesota Medical School

**Keywords:** ADAM10, EGR1, polymorphism, rs653765, sepsis

## Abstract

Increasing evidence has indicated that single nucleotide polymorphisms (SNPs) are related to the susceptibility of sepsis and might provide potential evidence for the mechanisms of sepsis. Our recent preliminary study showed that the ADAM10 genetic polymorphism was clinically associated with the development of sepsis, and little is known about the underlying mechanism. The aim of this study was to confirm the association between the ADAM10 promoter rs653765 G→A polymorphism and the progression of sepsis and to discover the underlying mechanism. Clinical data showed that the rs653765 G→A polymorphism was positively correlated with the development of sepsis, as evidenced by a multiple-center case-control association study with a large sample size, and showed that EGR1 and ADAM10 levels were associated well with the different subtypes of sepsis patients. *In vitro* results demonstrated that the rs653765 G→A variants could functionally modulate ADAM10 promoter activity by altering the binding of the EGR1 transcription factor (TF) to the ADAM10 promoter, affecting the transcription and translation of the *ADAM10* gene. Electrophoretic mobility shift assay (EMSA) followed by chromatin immunoprecipitation (ChIP) assay indicated the direct interaction. Functional studies further identified that the EGR1/ADAM10 pathway is important for the inflammatory response. EGR1 intervention *in vivo* decreased host proinflammatory cytokine secretion and rescued the survival and tissue injury of the mouse endotoxemia model.

## Abstract (precis)

Sepsis is characterized as life-threatening organ dysfunction, with unacceptably high mortality. Evidence has indicated that functional SNPs within inflammatory genes are associated with susceptibility, progression, and prognosis of sepsis. These mechanisms on which these susceptible sites depended often suggest the key pathogenesis and potential targets in sepsis. In the present study, we confirmed that a functional variant acts as an important genetic factor that confers the progression of sepsis in a large sample size and in multiple centers and revealed that the variants modulate the EGR1/ADAM10 pathway and influence the severity of sepsis. We believe that we provide an important insight into this new pathway involving the regulation of inflammatory process of sepsis based on the clinical genetic evidence, which will enhance the understanding of nosogenesis of sepsis and provide the potential target for inflammation-related diseases.

## INTRODUCTION

Sepsis is characterized as life-threatening organ dysfunction caused by a dysregulated host response to infection (1). Despite progress in the development of antibiotics and other optimal care therapies, sepsis remains the major cause of death in intensive care units (ICUs), with an associated mortality rate ranging from 30 to 70% (2–4). As a polygenic and multifactorial symptom, the etiology of sepsis is poorly characterized, and genetic mutations, inflammatory disorders, and dysregulation of metabolic homeostasis are the main risk factors for the susceptibility and progression of sepsis (5–7). Increasing evidence has indicated that functional single nucleotide polymorphisms (SNPs) within inflammatory-related genes are related to the susceptibility, development, and outcome of sepsis (8–15). These genetic variants may help to explain why the existence of genetic diversity caused interindividual differences in clinical outcomes of sepsis patients despite the same standardized treatment, and they also provide important clues for new mechanisms of sepsis.

A disintegrin and metalloproteases (ADAMs) are cell membrane-associated enzymes responsible for the liberation of a variety of cell surface-expressed proteins (16–18). Within the ADAM family, ADAM10 is one of the most widely expressed and extensively studied members and has been shown to be involved in the cleavage of more than 40 substrates, such as cytokines, chemokines, and adhesion molecules, and this cleavage process emerged as a key regulator in many biological processes ranging from nervous system development, immunity, and inflammatory disease (19–21). To date, cumulative evidence indicates that as a biologically multifunctional protease, ADAM10 plays a critical role in the inflammatory response (22–24), and targeting ADAM10 by conditional gene knockout or pharmacological inhibition not only attenuates the inflammatory response in animal models but also improves the outcome of infectious disease in humans (24–29). In recent years, several studies revealed the relationship between SNPs within the ADAM10 genes and the risk of inflammation-related diseases (30–34), while the mechanism underlying this important clinical evidence was unclear. Our previous studies initially indicated that the ADAM10 promoter polymorphism of rs653765 C→T was positively associated with the development of sepsis and showed evidence that rs653765 polymorphism may be a potential functional SNP for sepsis risk, yet how ADAM10 promoter polymorphism exerts effects on sepsis pathological remained unclear.

In the present study, we confirmed this positive association in a multiple-center case-control association study with a large sample size and discovered the variant-dependent underlying molecular mechanism responsible for this conferred correlation. Our results provide a new pathological EGR1/ADAM10 pathway in sepsis based on the exact genetic association: the rs653765 G→A variants modulate ADAM10 promoter activity by altering binding of the EGR1 transcription factor (TF) to the ADAM10 promoter and then functionally activate ADAM10 gene expression, concomitantly elevating relevant substrates. Further, EGR1 intervention decreased host proinflammatory cytokine production level and rescued the survival of the mouse endotoxemia model.

## RESULTS

### Baseline characteristics of participants.

In total, 1,025 sepsis patients and 1,152 healthy controls were consecutively recruited from three representative regions of China. The geographical distribution and demographic characteristics of sepsis patients and healthy controls are summarized in Table S1 in the supplemental material. The clinical characteristics and laboratory data of the cases are presented in Table S2. Numbers of patients suffering from mild sepsis, severe sepsis, and septic shock were 160, 507, and 358, respectively.

10.1128/mBio.01663-19.4 TABLE S1 Geographical distribution and demographic characteristics of participants. Download Table S1, DOCX file, 0.01 MB. Copyright © 2019 Chen et al. 2019 Chen et al. This content is distributed under the terms of the Creative Commons Attribution 4.0 International license.

10.1128/mBio.01663-19.5 TABLE S2 Baseline characteristics of sepsis cohort. Download Table S2, DOCX file, 0.02 MB. Copyright © 2019 Chen et al. 2019 Chen et al. This content is distributed under the terms of the Creative Commons Attribution 4.0 International license.

### Association between ADAM10 polymorphisms and the susceptibility and progression of sepsis.

Significant differences in genotype and allele frequency distributions were detected between the mild sepsis and severe sepsis subtypes, the mild sepsis and septic shock subtypes, and the mild sepsis and severe sepsis/septic shock subtypes (Table 1). Those findings suggest that the GG genotype and the G allele of the rs653765 are consistently associated with an increased risk for severe sepsis. No significant differences in genotypes or allele frequencies were observed between the sepsis patients and healthy individuals (Table S3), which corresponds with our previous results showing that the rs653765 polymorphisms of ADAM10 may not affect sepsis risk.

**TABLE 1:** Frequency distribution of rs653765 genotypes and alleles among sepsis subtypes^*a*^

rs653765 G→A genotype	No. (%) with:				*P1*	*P2*	*P3*	P1*	P2*	P3*
	Mild sepsis	Severe sepsis	Septic shock	Severe sepsis/septic shock						
All subjects	160	507	358	865						
GG	93 (58.1)	392 (78.9)	234 (70.5)	626 (75.5)	0.0001	0.0065	0.0001	0.0001	0.0065	0.0001
AG/AA	67 (41.9)	105 (21.1)	98 (29.5)	203 (24.4)						
G	241 (75.3)	878 (88.3)	560 (84.3)	1,438 (86.7)	0.0001	0.0007	0.0001	0.0001	0.0014	0.0001
A	79 (24.7)	116 (11.7)	104 (31.3)	220 (13.3)						
Zhanjiang	102	233	194	427						
GG	66 (64.7)	178 (76.4)	134 (69.1)	312 (73.1)	0.027	0.446	0.093	0.027	0.446	0.093
AG/AA	36 (35.3)	55 (23.6)	60 (30.9)	115 (26.9)						
G	162 (79.4)	402 (86.3)	323 (83.2)	725 (84.9)	0.025	0.249	0.056	0.027	0.446	0.093
A	42 (20.6)	64 (13.7)	65 (16.8)	129 (15.1)						
Harbin	43	249	93	342						
GG	19 (44.2)	197 (79.1)	71 (76.3)	268 (78.4)	0.0001	0.0002	0.0001	0.0001	0.0002	0.0001
AG/AA	24 (55.8)	52 (20.9)	22 (23.7)	74 (21.6)						
G	57 (66.3)	442 (88.8)	163 (87.6)	605 (88.5)	0.0001	0.0001	0.0001	0.0001	0.0002	0.0001
A	29 (33.7)	56 (11.2)	23 (12.4)	79 (11.5)						
Wuhan	15	25	71	96						
GG	8 (53.3)	22 (88.0)	44 (62.0)	66 (68.8)	0.014	0.534	0.239	0.014	0.534	0.239
AG/AA	7 (46.7)	3 (12.0)	27 (38.0)	30 (31.3)						
G	22 (73.3)	47 (94.0)	114 (80.3)	161 (83.9)	0.009	0.395	0.159	0.014	0.395	0.239
A	8 (26.7)	3 (6.0)	28 (19.7)	31 (16.1)						

a *P1*, mild sepsis versus severe sepsis; *P2*, mild sepsis versus septic shock; *P3*, mild sepsis versus severe sepsis and septic shock. An asterisk indicates false-discovery-rate-adjusted *P* value for multiple-hypothesis testing using the Benjamin-Hochberg method.

10.1128/mBio.01663-19.6 TABLE S3 Frequency distribution of rs653765 genotypes and alleles in cases and controls. Download Table S3, DOCX file, 0.02 MB. Copyright © 2019 Chen et al. 2019 Chen et al. This content is distributed under the terms of the Creative Commons Attribution 4.0 International license.

### Screening of TFs and influence of TFs on ADAM10 expression *in vitro*.

**FIG 1:**
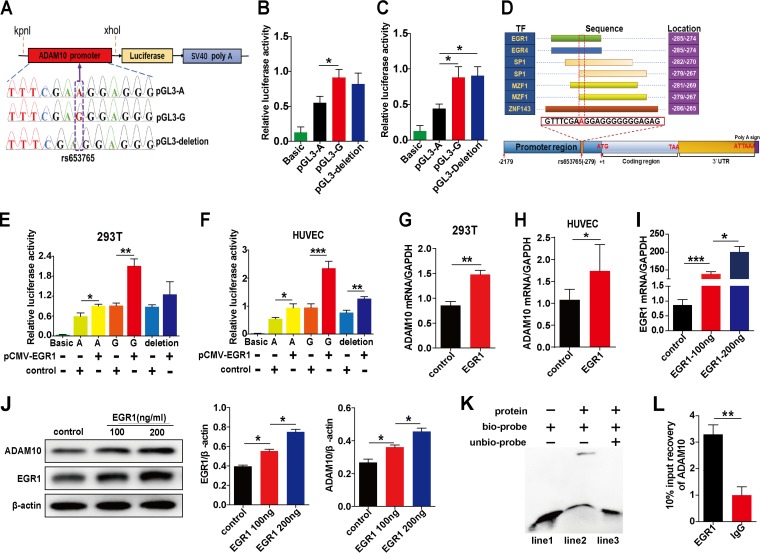
Screening of TFs and influence of TFs on ADAM10 expression *in vitro*. The ADAM10 promoter plasmids containing polymorphism of rs653765 (A, G, or 1-bp deletion) were cloned upstream of firefly luciferase in the pGL3-basic reporter plasmid (A). HEK-293T and HUVECs were transfected with pGL3-basic (1 μg/ml) as a baseline control or with different types of ADAM10 promoter luciferase reporter constructs (pGL3-A, pGL3-G, or pGL3-deletion) for 48 h to detect the effect of different genotype carriers on ADAM10 promoter reporter activity (B and C). (D) The prediction of TF that binds to the rs653765 site of the ADAM10 promoter. Functional effects of the predicted TFs EGR1 on ADAM10 promoter activity in HEK-293T and HUVECs were determined (E and F). The effects of EGR1 on ADAM10 mRNA expression were detected by qRT-PCR analysis (G and H). Expression levels of EGR1 were detected by qRT-PCR analysis after transfection with EGR1 plasmid for 48 h in cultured HUVECs (I). The effects of EGR1 on the expression of ADAM10 in cultured HUVECs for 72 h were detected by Western blot analysis (J). The interaction of EGR1 with the human ADAM10 promoter was detected by EMSA and ChIP assay. Nuclear EGR1 protein from HEK-293T was incubated with biotin-labeled DNA probes (ADAM10 promoter fragment) and subjected to EMSA by native PAGE (K). Lane 1, biotin-labeled probe only; lane 2, nuclear extracts plus biotin-labeled probe; lane 3, nuclear extracts plus biotin-labeled probe and cold unlabeled probe. The 100× unlabeled probe was used as a competitor for protein-DNA binding. The cleaved chromatin was incubated with an anti-EGR1 antibody and a negative-control IgG antibody. The result of EGR1 binding at the ADAM10 promoter was assessed by PCR amplification (L). The promoter activities were described as ratios of luciferase activities over *Renilla* luciferase activities. Data are presented as the means ± SEMs. At least three independent experiments were performed. *, *P* < 0.05; **, *P* < 0.01; ***, *P* < 0.001. SV40, simian virus 40.

**FIG 2:**
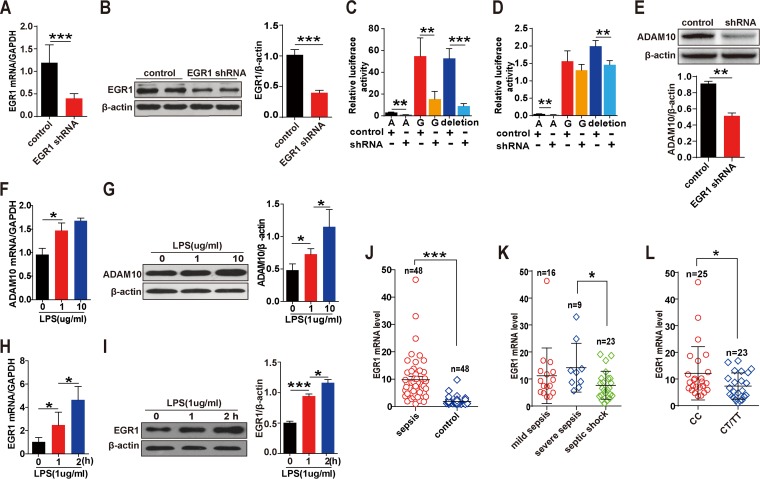
Distribution of EGR1 levels *in vitro* and *in vivo* under sepsis conditions. EGR1 shRNA and the control vectors (1 μg/ml) were transiently transfected into HUVECs for 48 h, and then the knockdown efficiency was detected by qRT-PCR (A) and Western blot analysis (B), respectively. ADAM10 promoter activity was detected by dual-luciferase reporter assays following 48 h of cotransfection of EGR1 shRNA or the negative-control plasmid (1 μg/ml) with the different types of ADAM10 promoter plasmids (1 μg/ml) in HEK-293T cells (C) and HUVECs (D). EGR1 shRNA or the negative-control plasmid (1 μg/ml) was transfected into HUVECs for 72 h, and the effect of EGR1 shRNA on ADAM10 expression was detected by Western blotting (E). The mRNA and protein expression levels of ADAM10 (F and G) and EGR1 (H and I) were detected by qRT-PCR and Western blot analysis. qRT-PCR analysis was used to determine the expression levels of EGR1 in PBMCs isolated from 48 sepsis patients and 48 matched healthy controls (J). EGR1 mRNA level distribution in the mild sepsis (*n* = 16), severe sepsis (*n* = 9) and septic shock (*n* = 23) subgroups was determined (K). Expression levels of EGR1 in sepsis patients based on genotype distribution were determined (L). Data are represented as the means ± SEMs. At least three independent experiments were performed. *, *P* < 0.05; **, *P* < 0.01; ***, *P* < 0.001.

The effect of the rs653765 polymorphism on the transcription activity of the *ADAM10* gene was examined. Remarkably higher luciferase expression was obtained with the construct carrying the G allele than with that of the A allele in both HEK-293T cells and human umbilical vascular endothelial cells (HUVECs) (Fig. 1A to C). The results suggest that the rs653765 polymorphism may be functional and may play pivotal roles in modulating ADAM10 promoter activity. Promoters are key regions for modulation gene transcriptions. Therefore, an rs653765 variant has the potential to alter the binding affinity of TFs. Five TFs, EGR1, EGR4, SP1, MZF1 and ZNF143, that blocked binding in the region surrounding rs653765 were predicted (Fig. 1D). Cells cotransfected with EGR1 displayed much higher luciferase activity than the cells transfected with vectors containing either the A or G allele, with a greater extent in the G allele group than in the A allele group (Fig. 1E and F). With regard to the other TFs, the difference did not show an obvious increase over the amount with EGR1 (Fig. S1A to H). These results indicate that the transcription of the ADAM10 promoter could be enhanced by EGR1 in an allele-dependent manner. Next, overexpression of EGR1 showed significantly higher ADAM10 mRNA levels (Fig. 1G and H); transfection with other vectors did not show a significant increase (Fig. S1I and J). Furthermore, compared to the other TFs, overexpressed EGR1 resulted in significantly increased ADAM10 protein levels in a dose-dependent manner (Fig. 1I and J; see also Fig. S1K to R). Moreover, electrophoretic mobility shift assay (EMSA) and chromatin immunoprecipitation (ChIP) assay determined the direct interaction between the EGR1 protein and the promoter sequence of the *ADAM10* gene in HEK-293T cells (Fig. 1K and L). Then we further evaluated the influence of the EGR1 short hairpin RNA (shRNA) on the expression level of ADAM10. The EGR1 mRNA and protein levels were significantly decreased in HUVECs (Fig. 2A and B), suggesting the knockdown efficiency of the shRNA. The EGR1 shRNA significantly reduced the luciferase expression in HEK-293T cells and HUVECs (Fig. 2C and D). Furthermore, the ADAM10 protein level was significantly attenuated after transfection with EGR1 shRNA (Fig. 2E). These results combined with above-described dual-luciferase reporter assay results confirmed that EGR1 is a candidate TF in the regulation of the *ADAM10* gene.

10.1128/mBio.01663-19.1 FIG S1 Screening of TFs and the Influence of TFs on ADAM10 expression *in vitro*. Download FIG S1, DOCX file, 0.3 MB. Copyright © 2019 Chen et al. 2019 Chen et al. This content is distributed under the terms of the Creative Commons Attribution 4.0 International license.

### Distribution of EGR1 levels in sepsis patients.

The expression level of EGR1 in sepsis patients was evaluated to further characterize the clinical association between EGR1 and ADAM10. Data showed that patients displayed a significantly higher EGR1 mRNA level than healthy controls (Fig. 2J). Subgroup analysis found that the EGR1 expression level was significantly increased in severe sepsis compared with that in the septic shock group (Fig. 2K). Next, the genotype distribution results showed that the rs653765 GG carriers exhibited substantially higher EGR1 mRNA levels than the patients who carried the AA or GA genotype (Fig. 2L). In addition, the expression levels of ADAM10 and EGR1 mRNA and protein levels were significantly upregulated in lipopolysaccharide (LPS)-exposed HUVECs (Fig. 2F to I). This evidence suggests the potential crucial role of EGR1 in regulating ADAM10 expression in sepsis patients.

### Influence of EGR1 on levels of related cytokines, adhesion molecules, and cell apoptosis.

**FIG 3:**
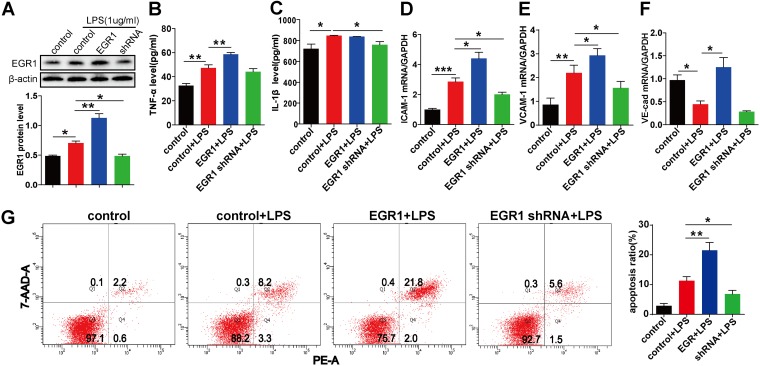
Effect of EGR1 on the expression levels of related cytokines, adhesion molecules, and cell apoptosis. HUVECs were transfected with 1 μg/ml of EGR1, shRNA, and control plasmid for 24 h, followed by incubation with LPS (1 μg/ml) for 24 h. Total protein and RNA were extracted, supernatant samples were harvested, and cell apoptosis was detected. The protein expression of EGR1 was detected by Western blot analysis (A). The concentrations of TNF-α (B) and IL-1β (C) were determined by ELISA. The expression levels of ICAM-1 (D), VCAM-1 (E), and VE-cadherin (F) mRNA were detected by qRT-PCR analysis. The apoptosis of HUVECs was evaluated by annexin V-PE/7-AAD staining assay and flow cytometry (G). The results of qRT-PCR and Western blot analyses are presented as multiple of the control value. ELISA data represent the mean concentrations of the related cytokines in each group. Data are presented as the means ± SEMs. At least three independent experiments were performed. *, *P* < 0.05; **, *P* < 0.01; ***, *P* < 0.001.

To determine whether EGR1 further affects the expression levels of ADAM10 substrates and the inflammatory process, we evaluated the correlation between EGR1 administration and the expression levels of related proinflammatory cytokines and endothelial adhesion molecules in LPS-activated HUVECs. The EGR1 protein levels were significant influenced by overexpression of EGR1 and the shRNA plasmid (Fig. 3A). Treatment with EGR1 significantly exacerbated LPS-induced secretion of tumor necrosis factor alpha (TNF-α) in HUVECs, and silencing of EGR1 by specific shRNA ameliorated the interleukin 1β (IL-1β) expression level (Fig. 3B and C). For the adhesion molecules, significantly increased ICAM-1, VCAM-1, and VE-cad mRNA expression levels were found with treatment with EGR1, and the shRNA administration was associated with significantly decreased ICAM-1 and VCAM-1 mRNA expression in LPS-stimulated HUVECs (Fig. 3D to F). Furthermore, overexpression of EGR1 resulted in significantly higher endothelial cell apoptosis, and the inhibition of EGR1 led to a markedly decreased apoptosis ratio compared with that obtained with LPS stimulation alone (Fig. 3G). Taken together, these findings substantiated that the EGR1/ADAM10 signaling pathway is a key mediator involved in the regulation inflammatory process *in vitro*.

### EGR1 As-ODN rescued the survival and tissue injury in an LPS-driven mouse endotoxemia model.

**FIG 4:**
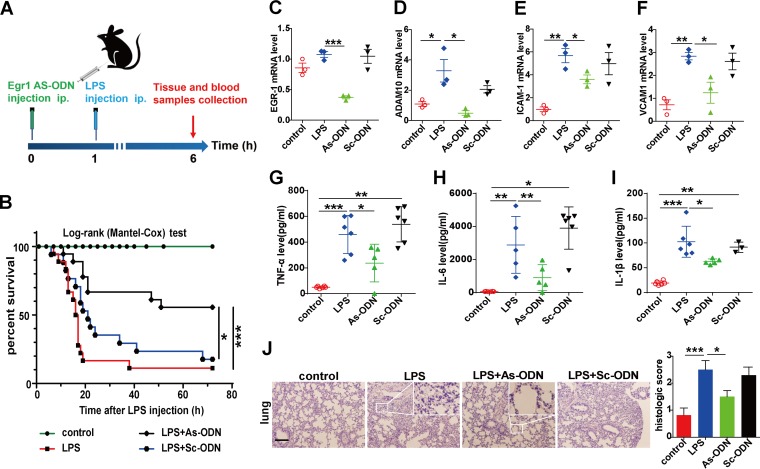
EGR1 As-ODN protects against the sepsis mouse model. Shown is a schematic diagram of the experimental procedures (A). Mice were treated with As-ODN, Sc-OND, or a vehicle via intraperitoneal injection for 1 h before LPS or normal saline was administered via intraperitoneal injection for 6 h. Then the mice were anesthetized, and vein blood samples and lung tissues were separated for further analysis. After the LPS (18 mg/kg) procedure, the survival rate was monitored continuously throughout the experiment, and the data were analyzed by a Kaplan-Meier survival curve (*n* = 17 for control and Sc-OND groups and *n* = 18 for LPS and As-ODN groups) (B). The mRNA levels of EGR1 (C), ADAM10 (D), ICAM-1 (E), and VCAM-1 (F) in lung tissues were determined by qRT-PCR. Blood samples were isolated 6 h after LPS injection, and serum was analyzed by ELISA for TNF-α (G), IL-6 (H), and IL-1β (I) release. Histological changes of lung tissue sections from each group were stained with hematoxylin and eosin (H&E) (J). Scale bars, 100 μm. Images were taken at a magnification of ×100. Data are presented as the means ± SEMs (at least 3 animals per group). *, *P* < 0.05; **, *P* < 0.01; ***, *P* < 0.001.

Finally, whether EGR1 intervention modulates host survival and tissue injury in an LPS-mediated mouse endotoxemia model was evaluated (Fig. 4A). Pre-experiment data showed that LPS at 15 mg/kg of body weight was the most effective and stable dose in promoting proinflammatory cytokine secretion (Fig. S2A to C). In the survival experiment, administration with EGR1 antisense ODN (As-ODN) significantly rescued the survival rate of the mice (Fig. 4B). Tissue injury was evaluated by hematoxylin and eosin (H&E) staining, and As-ODN significantly eliminated the pathological alterations (Fig. 4J and Fig. S2O). Increasing levels of EGR1 in kidney, lung, and liver tissues were rapidly elicited in response to LPS, peaking at 30 min post-LPS stimulation (Fig. S2D to F). Compared to that in the scrambled-sequence ODN (Sc-ODN) group, the EGR1 mRNA level was significantly reduced in the lung tissues of mice with LPS-induced sepsis, suggesting the knockdown efficiency of As-ODN (Fig. 4C), and consistent results were also observed in liver and kidney tissues with the intervention of As-ODN (Fig. S2G and K). Furthermore, the mRNA expression of ADAM10 was significantly downregulated in the EGR1 As-ODN group within lung (Fig. 4D) and kidney and liver tissues (Fig. S2H and L), which was consistent with our *in vitro* results. ADAM10 acts as an EGR1 target gene in the mouse endotoxemia model. Moreover, the concentrations of the proinflammatory factors (TNF-α, IL-6, and IL-1β) and adhesion molecules (ICAM-1 and VCAM-1) were significantly downregulated by As-ODN treatment as evoked by LPS (Fig. 4E to I and Fig. S2I, J, M, and N). In conclusion, inhibition of EGR1 effectively attenuated tissue injury and improved survival in an LPS-driven endotoxemia model in mice.

10.1128/mBio.01663-19.2 FIG S2 EGR1 As-ODN protects against the sepsis mouse model. Download FIG S2, DOCX file, 0.6 MB. Copyright © 2019 Chen et al. 2019 Chen et al. This content is distributed under the terms of the Creative Commons Attribution 4.0 International license.

## DISCUSSION

**FIG 5:**
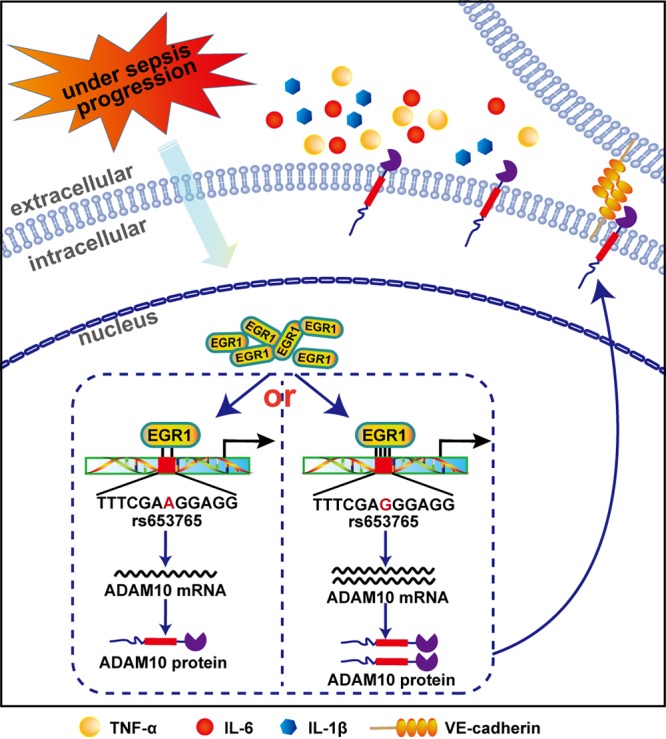
Mechanisms of rs653765 G→A associated with sepsis progression. Shown is a diagram illustrating that the polymorphism of rs653765 G→A-mediated disruption of EGR1 binding at the promoter region of the ADAM10 gene functionally activates ADAM10 gene expression, triggering sepsis progression.

We confirmed that the ADAM10 promoter rs653765 G→A polymorphism contributes to the progression of sepsis in a multiple-center case-control association study with a large sample size, and we discovered the molecular mechanism underlying the functional modulation of ADAM10 promoter activity by the rs653765 G→A variant. The direct binding of the EGR1 to the ADAM10 promoter is modified, affecting the transcription and translation of the *ADAM10* gene, resulting in enhanced inflammatory responses and ultimately stimulating the progression of sepsis *in vitro* and *in vivo* (Fig. 5).

ADAM10 is a ubiquitously expressed zinc metalloprotease that is involved in the regulation of cellular adhesion and systematic inflammation through the cleavage of dozens of substrates (17, 35, 36). Studies have demonstrated that the expression levels of ADAM10 increased in various murine models of sepsis, which could reflect the organ dysfunction and mortality seen in sepsis (26, 37). The rs653765 polymorphism, located in the ADAM10 promoter region, widely exists in different regions and among different races in the world. Our study confirmed that the rs653765 G→A SNP contributes to sepsis progression in a large sample size of sepsis patients recruited from three representative areas of China, which authenticated the susceptibility gene of ADAM10 and added a new mutation site for sepsis.

The underlying regulatory mechanisms by which the promoter rs653765 G→A SNP confers sepsis progression have not yet been elucidated. As a *cis*-acting element, the promoter is the center for orchestrating gene transcription due to harboring numerous TF binding sites (38–40). Genetic variations in the promoter regions have been demonstrated to modify the transcriptional activity of target genes by altering the binding affinity of TFs and further influence the biological function of target genes (41–43). We hypothesized that the allelic variation at the rs653765 site interferes with *ADAM10* gene translation efficiency. As expected, remarkably higher luciferase expression was observed with the construct carrying the sepsis-associated G risk allele of rs653765 in comparison with that of the construct with the A allele, suggesting that this SNP may be functional and play pivotal roles in regulating *ADAM10* gene transcription.

To assess which TFs affect ADAM10 responsiveness, bioinformatics analysis identified that rs653765 lies in the transcription recognition region of EGR1, EGR4, SP1, MZF1, and ZNF143. Dual-luciferase reporter data coupled with quantitative reverse transcription-PCR (qRT-PCR) and Western blot assays collectively implied a potential role for EGR1 as a major regulator involved in the regulation of ADAM10 expression. Studies that identified several known EGR1 target genes suggested that most of the genes were regulated directly, and the binding domains were present in promoter regions; however, no evidence has shown that ERG1 is the TF of ADAM10. EMSA followed by the ChIP assay indicate that *ADAM10* is a direct target gene of EGR1, further adding to the chain of proof that the rs653765 variant alters *ADAM10* gene transcription and expression by regulating the binding affinity of EGR1 with the ADAM10 promoter domains.

EGR1 (also known as NGFI-A, krox-24, Zif268, or TIS8) encodes a Cys2-His2-type zinc finger TF that belongs to the member of the immediate early gene family, and its expression is rapidly elicited in response to a variety of stimuli (44). Once activated, EGR1 may function as a master switch to trigger the expression of numerous downstream target genes and plays pivotal roles in various cellular programs, including those involved in inflammation events and cell apoptosis (45–47). Here we show that patients with sepsis exhibited significantly higher EGR1 mRNA expression levels than the healthy controls. Consistently, our previous study showed that the ADAM10 mRNA expression levels in the sepsis group were significantly higher than in controls (33), underscoring the fact that EGR1 correlates well with ADAM10 expression in sepsis patients and potentially plays a crucial role in the progression of sepsis. Furthermore, the rs653765 GG genotype carriers displayed significantly higher levels of EGR1 than the GA/AA carriers. Notably, our previous study confirmed that the rs653765 GG genotype carriers exhibited higher ADAM10 levels as well as increased secretion of the ADAM10 substrates (33). These results suggest that the rs653765 SNP might impact the EGR1/ADAM10 signaling pathway and ultimately contribute to the progression of sepsis.

Emerging evidence suggests that the biological effects of ADAM10 activity are tightly linked to the development and progression of inflammatory response via various cellular processes, which may represent key regulatory mechanisms regarding sepsis progression. Therefore, we determined the functional effects of the EGR1/ADAM10 pathway in regulation-related cytokine production and adhesion molecule expression under LPS exposure. The results showed that treatment with or genetic inhibition of EGR1 triggered significant effects on cytokine and adhesion molecule expression, which further validated the functional pathway of EGR1/ADAM10 in the context of endotoxemia. Apoptotic processes are of substantial importance in the course of systemic inflammation and are critical markers for sepsis (48–50). We showed that EGR1 significantly increased endothelial cell apoptosis and that the inhibition of EGR1 resulted in a markedly decreased apoptosis ratio under LPS stimulation, which is in line with previous reports. Thus, our data suggest new insight into the novel mechanism of the EGR1/ADAM10 pathway in the regulation of sepsis progression.

EGR1 is recognized as a pleiotropic inflammatory transactivator that appears to be a master regulator in a variety of proinflammatory pathological processes, which is closely associated with the pathogenic mechanism of inflammatory-related diseases, such as sepsis and cancer (51–55). Evidence has confirmed the critical role of EGR1 in regulating the inflammatory response, while the specific mechanisms remain unclear (54, 56). In light of the crucial role of the EGR1/ADAM10 signaling pathway in the sample of sepsis patients and *in vitro* models that confirmed this, we evaluated whether EGR1 could serve as a therapeutic target for sepsis. Our *in vivo* results showed that inhibition of EGR1 by specific As-ODN was associated with decreased ADAM10 expression with various efficiencies in kidney, liver, and lung tissues and was accompanied by reduced circulating proinflammatory marker generation and expression of tissue adhesion molecules, which ultimately rescued the survival and tissue injury of mice with endotoxemia. Collectively, these data provide insights into the mechanisms showing that targeting the EGR1/ADAM10 pathway may be a potential therapeutic regimen for mice with endotoxemia driven by LPS.

In summary, we corroborated that the functional variant of rs653765 acts as an important genetic factor that confers the progression of sepsis in a large cohort and revealed that the rs653765 G→A variants modulate ADAM10 promoter activity by altering the binding of the EGR1 to the ADAM10 promoter, which then functionally activates ADAM10 gene expression and may influence the prognosis of sepsis. Our study reports the mechanism of the association between rs653765 and the risk of sepsis progression and provides the EGR1/ADAM10 pathway involving the regulation of nosogenesis of sepsis based on the clinical genetic evidence. Additional studies will shed light on the role of this pathway in the pathogenesis of sepsis and further clarify its prognostic and therapeutic potential.

## MATERIALS AND METHODS

### Study population.

A total of 1,025 patients with confirmed sepsis and 1,152 matched healthy controls were consecutively recruited from three representative regions of China between July 2012 and May 2018. The sepsis subtypes were diagnosed according to the International Sepsis Definitions Conference (4). The clinical studies mainly include the process of subject enrollment, primary analysis, and subgroup analysis (Fig. S3). Genomic DNA extraction and genotyping were performed as previously described (33). Each participant or their legal representatives provided informed consent for the study. The protocols employed in this research were approved by the Ethics Committee of the Affiliated Hospital of Guangdong Medical University, Center Hospital of Wuhan, and Harbin Medical University.

10.1128/mBio.01663-19.3 FIG S3 Flowchart of subject inclusion into clinical analysis cohort. Download FIG S3, DOCX file, 0.2 MB. Copyright © 2019 Chen et al. 2019 Chen et al. This content is distributed under the terms of the Creative Commons Attribution 4.0 International license.

### Cell culture.

Human umbilical vascular endothelial (HUVEC) and human embryonic kidney (HEK-293T) cell lines were purchased from Shanghai Institute of Cell Biology (Shanghai, China). HUVECs and HEK-293T cells were maintained in Dulbecco’s modified Eagle's medium (DMEM, Gibco, USA) and RPMI 1640 medium, respectively. Culture media were supplemented with 10% fetal bovine serum (FBS; Gibco, USA), 100 U/ml of penicillin, and 100 mg/ml streptomycin and incubated at 37°C in a humidified atmosphere containing 5% CO_2_.

### Plasmid construction, TF prediction, transfection, and luciferase reporter assay.

ADAM10 promoter fragments (2,482 bp, from bp −2000 to +482 in relation to the transcription start site) were synthesized by PCR amplification and inserted into the KpnI and XhoI sites of pGL3-basic to construct expression vectors (pGL3-A, pGL3-G, and pGL3-deletion). The PCR primer sequences with KpnI or XhoI sites were as follows: 5′-ATAGGTACCTAGCAGAGACGGAGTTTCACC-3′ (forward) and 5′-AATAACTCGAGCAGGAGAGGAGCAGAATTAACAC-3′ (reverse). The potential TFs were predicted by TFBIND (http://tfbind.hgc.jp). Cells were transfected with different types of ADAM10 promoter expression vectors or cotransfected with each TF plasmid (pCMV-EGR1, pCMV-EGR4, pCMV-SP1, pCMV-MZF1, pCMV-ZNF143, and empty control) by Lipofectamine 2000 (Invitrogen, USA) according to the manufacturer’s instructions. Dual-luciferase activities were detected by the dual-luciferase assay kit (Promega, USA), and luminescence was measured using a Mithras LB940 multilabel reader (Berthold Technologies, Bad Wildbad, Germany).

### qRT-PCR and Western blot analysis.

Peripheral blood mononuclear cells (PBMCs) were isolated from peripheral blood for qRT-PCR analysis as previously described (33); primers designed in this study are listed in Table S4. The proteins were separated by 10% sodium dodecyl sulfate-polyacrylamide gel electrophoresis (SDS-PAGE) and incubated with a specific antibody (anti-ADAM10, diluted 1:500, ab53281 [Abcam, Cambridge, UK], anti-EGR1, diluted 1:800, sc-13943 [Santa Cruz Biotechnology], and anti-β-actin, diluted 1:2,000 [Santa Cruz Biotechnology]) at 4°C overnight, followed by horseradish peroxidase-conjugated goat anti-rat IgG as the secondary antibody. A chemiluminescence detection kit (catalog no. WBKLS0100; Millipore, USA) was used to visualize immunoreactive bands.

10.1128/mBio.01663-19.7 TABLE S4 Primer sequences used for quantitative real-time PCR analysis. Download Table S4, DOCX file, 0.02 MB. Copyright © 2019 Chen et al. 2019 Chen et al. This content is distributed under the terms of the Creative Commons Attribution 4.0 International license.

### Electrophoretic mobility shift assay.

Nuclear extracts were prepared from the HEK-293T cells and EMSA was performed by using the LightShift chemiluminescent EMSA kit (Pierce, Rockford, IL). A biotin-labeled double-stranded oligonucleotide (5′-GAGGTCTGAGTTTCGAAGGAGGGGGGGAGAG-3′) containing a consensus EGR1 motif was used as the EMSA probe. An unlabeled double-stranded oligonucleotide was used as a competitor probe. Anti-EGR1 antibody (catalog no. 4154S; CST) was used to supershift the DNA-protein complex. For the binding reaction system, 6 μl of master mix, 5 μg of protein, 2 μl of biotin-labeled probe, 2 μl of unlabeled probe, 2 μl of mutant probe, and 1 μl of antibody were added. For competition studies, unlabeled double-stranded oligonucleotides (100-fold molar excess) were used. The reaction products were incubated for 20 min on ice and separated in 4% nondenaturing polyacrylamide gels.

### Chromatin immunoprecipitation assay.

HEK-293T cells were cross-linked with 1% formaldehyde for 10 min at room temperature, washed with ice-cold phosphate-buffered saline (PBS), and stop-fix solution containing glycine (125 mM), aprotinin (1 μg/ml), pepstatin A (1 μg/ml) and Pefabloc (1 mg/ml) was added to neutralize the DNA-protein cross-linking for 5 min at room temperature. The samples were pelleted by centrifugation at 2,500 × *g* and 4°C for 2 min and washed with ice-cold PBS. Chromatin was immunoprecipitated with immunoglobulin IgG (Sigma) and anti-EGR1 (catalog no. 4154S; CST). The association of EGR1 with ADAM10 was measured by qRT-PCR with the following primers: forward, 5′-CAGGCCTAGCAGCACGGGA-3′, and reverse, 5′-TCCCTTGCTCGTTCCCTCT-3′.

### Annexin V apoptosis assay.

An annexin V-phycoerythrin (PE)/7-aminoactinomycin D (7-AAD) staining apoptosis detection kit (Millipore, MA, USA) was used to analyze the apoptosis of HUVECs according to the manufacturer’s protocols. Briefly, cells were collected and washed twice with ice-cold PBS and resuspended in 100 μl of binding buffer. Then 5 μl of annexin V/PE and 10 μl of 7-AAD were added to the cell samples, followed by incubation for 15 min in the dark at room temperature. After that, samples were analyzed by flow cytometry.

### EGR1 antisense ODN and scrambled-sequence ODN.

EGR1 As-ODN and Sc-ODN were synthesized (TaKaRa Biotechology Co., Ltd., China) as described previously (56). The sequences of As-ODN and Sc-ODN were 3′-TACCGTCGCCGGTTC-5′ and 3′-TCGTGCCGCTGCCAT-5′, respectively.

### Mouse administration.

Female C57BL/6 mice (6 to 8 weeks, weighing 20 to 25 g) were obtained from the Laboratory Animal Center of Guangdong Medical University (Zhanjiang, China) and maintained at a controlled temperature of 24 to 26°C under a 12-h light/dark schedule. Mice had free access to food and purified water. All animal experimental procedures were performed in accordance with the *Guide for the Care and Use of Laboratory Animals* (57) and approved by the laboratory animal ethical committee of Guangdong Medical University (identifier [ID] number GDY1701017). Mice were randomly divided into four groups: control, lipopolysaccharide (LPS), As-ODN, and Sc-ODN. The As-ODN and Sc-ODN groups were intraperitoneally injected with As-ODN and Sc-ODN (20 mg/kg), respectively, and the control and LPS groups were injected with the equivalent volumes of saline. One hour after the injection, the mice in the LPS, As-ODN, and Sc-ODN groups were intraperitoneally challenged with LPS (Escherichia coli O55:B5, 15 mg/kg), and the control group received equivalent volumes of saline.

### Enzyme-linked immunosorbent assay.

The levels of cytokines (TNF-α, IL-6, and IL-1β) were assayed by specific commercial enzyme-linked immunosorbent assay (ELISA) kits (Beyotime Biotechnology, Shanghai, China) by following the manufacturer’s instructions. The data were calculated from standard curves of relevant cytokines using the linear regression method.

### Hematoxylin and eosin staining.

Six hours after LPS administration, mice were anesthetized and sacrificed. The kidney, lung, and liver tissues were removed, and parts of the tissues were incubated in 4% formalin overnight at 4°C, dehydrated by decreasing concentrations of ethanol, embedded in paraffin wax, and sectioned at a 5-μm thickness. Then slices were deparaffinized, rehydrated in decreasing concentrations of ethanol, and stained with H&E for semiquantitative histological analysis of tissue injury. Kidney tissue damage was evaluated as tubular epithelial swelling, vacuolar degeneration, necrotic tubules, cast formation, and desquamation. Liver injury was defined as the amount of destruction of hepatic lobules, hepatocyte necrosis, hemorrhage, and infiltration of inflammatory cells. Lung tissue damage was described as lung edema, hemorrhage, hyaline membrane, inflammatory cell infiltration, and atelectasis. Blind analysis was performed to determine the degree of lesions in all samples. All of the histological analysis used scoring from 1 through 4 according to percentage of area per field, and the sum of all scores was considered the total score on pathology of tissues.

### Statistical analysis.

The data were given as the means ± standard errors of the means (SEM) and compared using Student’s *t* test. Chi-square test or Fisher’s exact test was used to evaluate the allele and genotype frequencies between cases and controls as well as within the subgroups, and *P* values were further subjected to Bonferroni correction to account for multiple comparisons. Risk-associated genotypes were calculated using the odds ratio (OR) at a 95% confidence interval (CI) with a two-tailed level of significance. The Kaplan-Meier method was adopted to estimate survival curves. Statistical analyses were performed using the SPSS program (version 12.0; SPSS, Chicago, IL) and GraphPad Prism 5.0 (GraphPad Inc., San Diego, CA) software. Significance was established at a *P* value of <0.05.
