# Supplementary material for: A Functional Polymorphism-Mediated Disruption of EGR1/ADAM10 Pathway Confers the Risk of Sepsis Progression
Source: mBio. 2019 Aug 6;10(4):e01663-19. doi: 10.1128/mBio.01663-19 (PMC6686044; doi:10.1128/mBio.01663-19)
Supplement: TABLE S1 [file mBio.01663-19-st001.docx]

**Table S1. Geographical distribution and demographic characteristics of participant**

| Demographics | Sepsis (n) | Control (n) | *P* value |
| --- | --- | --- | --- |
| **All subjects** | 1025 | 1152 |  |
| Age, mean ± SD | 61.6 ± 1.7 | 48.3 ± 1.4 | 0.001 |
| Male/female, number | 621/404 | 645/507 | 0.030 |
| **Zhanjiang (In southern China)** | 529 | 610 |  |
| Age, mean ± SD | 61.1 ± 2.2 | 48.4 ± 2.4 | 0.001 |
| Male/female, number | 339/190 | 331/279 | 0.001 |
| **Harbin (In northern China)** | 385 | 403 |  |
| Age, mean ± SD | 60.8 ± 1.8 | 48.4 ± 1.5 | 0.001 |
| Male/female, number | 209/176 | 226/177 | 0.613 |
| **Wuhan (In central China)** | 111 | 139 |  |
| Age, mean ± SD | 65.6±1.5 | 45.3±1.2 | 0.001 |
| Male/female, number | 73/38 | 88/51 | 0.687 |

Demographic characteristics of sepsis patients and healthy controls. Significant differences in age or gender distribution were detected between the cases and the controls.
