# Supplementary material for: A Functional Polymorphism-Mediated Disruption of EGR1/ADAM10 Pathway Confers the Risk of Sepsis Progression
Source: mBio. 2019 Aug 6;10(4):e01663-19. doi: 10.1128/mBio.01663-19 (PMC6686044; doi:10.1128/mBio.01663-19)
Supplement: TABLE S2 [file mBio.01663-19-st002.docx]

**Table S2. Baseline Characteristics of sepsis cohort**

| **Variable** | **Mild sepsis n(%)** | **Severe sepsis**  **n(%)** | **Septic shock**  **n(%)** |
| --- | --- | --- | --- |
| **Sepsis status** | 160(15.6) | 507(49.5) | 358(34.9) |
| **Source of infection, n (%)** |  |  |  |
| Lung tract infection | 97(60.6) | 293(57.8) | 203(56.7) |
| Bloodstream infection | 11(6.9) | 47(9.3) | 39(10.9) |
| Abdominal infection | 13(8.1) | 36(7.1) | 27(7.5) |
| Urinary tract infection | 14(8.8) | 37(7.3) | 32(8.9) |
| Wound infection | 8(5.0) | 29(5.7) | 16(4.5) |
| Others | 17(10.6) | 65(12.8) | 41(11.5) |
| **Identified pathogen, n (%)** |  |  |  |
| Gram-negative | 62(38.8) | 214(42.2) | 156(43.6) |
| Gram-positive | 24(15.0) | 79 (15.6) | 47(13.1) |
| Mixed Gram-negative and -positive | 35(21.9) | 99(19.5) | 63(17.6) |
| Fungus | 15(9.4) | 34(6.7) | 27(7.5) |
| Polymicrobial | 15(9.4) | 29(5.7) | 26(7.3) |
| Negative blood culture | 9(5.6) | 52(10.3) | 39(10.9) |
| **Pathogen in blood culture, n (%)** |  |  |  |
| *Acinetobacter baumannii* | 31(19.4) | 109(21.5) | 84(23.5) |
| *Escherichia coli* | 18(11.3) | 53(10.4) | 33(9.2) |
| *Pseudomonas aeruginosa* | 12(7.5) | 35(6.9) | 31(8.7) |
| *Klebsiella pneumoniae* | 11(6.9) | 37(7.3) | 19(5.3) |
| *Staphylococcus aureus* | 10(6.3) | 31(6.1) | 27(7.5) |
| *Corynebacterium* | 13(8.1) | 30(5.9) | 17(4.7) |
| various bacteria | 46(28.8) | 139(27.4) | 84(23.5) |
| Other pathogens | 19(11.9) | 73(14.4) | 63(17.6) |
| **Base-line laboratory values** |  |  |  |
| Lactate (mmol/L) | 2.48±0.34 | 2.22±0.12 | 5.07±0.35 |
| Serum procalcitonin (ug/L) | 2.87±0.66 | 9.99±1.28 | 16.81±1.59 |
| Base-line WBC count (cell/mm^3^) | 15.35±1.24 | 17.91±0.70 | 17.67±0.62 |
| APACHE II score | 14.18±0.73 | 18.64±0.52 | 21.74±0.52 |

Abbreviations: APACHE II, Acute Physiology and Chronic Health Evaluation II; WBC, white blood cell. Continuous data are expressed as the mean ± SEM.

The most frequent site of infection was respiratory tract, followed by the bloodstream, urinary tract, abdominal tissue, wound infection and others. Gram-negative and Gram-positive bacteria and fungi were the primary pathogens.
