# Supplementary material for: A Functional Polymorphism-Mediated Disruption of EGR1/ADAM10 Pathway Confers the Risk of Sepsis Progression
Source: mBio. 2019 Aug 6;10(4):e01663-19. doi: 10.1128/mBio.01663-19 (PMC6686044; doi:10.1128/mBio.01663-19)
Supplement: TABLE S3 [file mBio.01663-19-st003.docx]

**Table S3. Frequency distribution of rs653765 genotypes and alleles in cases and controls**

| **Genotype rs653765 G > A** | **Sepsis n=1025 (%)** | **Control n=1152(%)** | ***P*** | ***P**** | **Adjusted odds**  **ratio (95% CI)** |
| --- | --- | --- | --- | --- | --- |
| **All subjects** | 1025 | 1152 |  |  |  |
| GG | 739(72.1) | 795(69.0) | 0.273 | 0.364 | - |
| AG | 254(24.8) | 314(27.3) | - | - | - |
| AA | 32(3.1) | 43(3.7) | - | - | - |
| GG/AG | 993(96.9) | 1109(96.3) | 0.435 | 0.223 | 1.203 (0.755, 1.917) |
| AA/AG | 286(27.9) | 357(31.0) | 0.115 | 0.230 | 0.862 (0.716, 1.037) |
| G | 1732(84.5) | 1904(82.6) | - | - | 1.000 (reference) |
| A | 318(15.5) | 400(17.4) | 0.101 | 0.230 | 1.144 (0.974, 1.344) |
| **Zhanjiang** | 529 | 610 |  |  |  |
| GG | 378(71.5) | 420(68.9) | 0.348 | 0.571 | - |
| AG | 131(24.8) | 172(28.2) | - | - | - |
| AA | 20(3.8) | 18(3.0) | - | - | - |
| GG/AG | 509(96.2) | 592(97.0) | 0.437 | 0.571 | 0.774 (0.405, 1.479) |
| AA/AG | 151(28.5) | 190(31.1) | 0.387 | 0.571 | 0.883 (0.684, 1.139) |
| G | 887(83.8) | 1012(83.0) | - | - | 1.000 (reference) |
| A | 171(16.2) | 208(17.0) | 0.571 | 0.571 | 1.066 (0.854, 1.331) |
| **Harbin** | 385 | 403 |  |  |  |
| GG | 287(74.5) | 281(69.7) | 0.085 | 0.113 | - |
| AG | 88(22.9) | 100(24.8) | - | - | - |
| AA | 10(2.6) | 22(5.5) | - | - | - |
| GG/AG | 375(97.4) | 381(94.5) | 0.042 | 0.084 | 2.165 (1.011, 4.636) |
| AA/AG | 98(25.5) | 122(30.3) | 0.132 | 0.132 | 0.787 (0.575, 1.075) |
| G | 662(86.0) | 662(82.1) | - | - | 1.000 (reference) |
| A | 108(14.0) | 144(17.9) | 0.038 | 0.084 | 1.333 (1.016, 1.750) |
| **Wuhan** | 111 | 139 |  |  |  |
| GG | 74(66.7) | 94(67.6) | 0.9597 | 1.000 | - |
| AG | 35(31.5) | 42(30.2) | - | - | - |
| AA | 2(1.8) | 3(2.2) | - | - | - |
| GG/AG | 109(98.2) | 136(97.8) | 1.000 | 1.000 | 1.202(0.197, 7.33) |
| AA/AG | 37(33.3) | 45(32.4) | 0.893 | 1.000 | 1.044 (0.614, 1.776) |
| G | 183(82.4) | 230(82.7) | - | - | 1.000 (reference) |
| A | 39(17.6) | 48(17.3) | 1.000 | 1.000 | 0.9793 (0.615, 1.559) |

*False discovery rate-adjusted P-value for multiple hypotheses testing using the Benjamin-Hochberg method.
