# Supplementary material for: A Functional Polymorphism-Mediated Disruption of EGR1/ADAM10 Pathway Confers the Risk of Sepsis Progression
Source: mBio. 2019 Aug 6;10(4):e01663-19. doi: 10.1128/mBio.01663-19 (PMC6686044; doi:10.1128/mBio.01663-19)
Supplement: FIG S1 [file mBio.01663-19-sf001.docx]

| 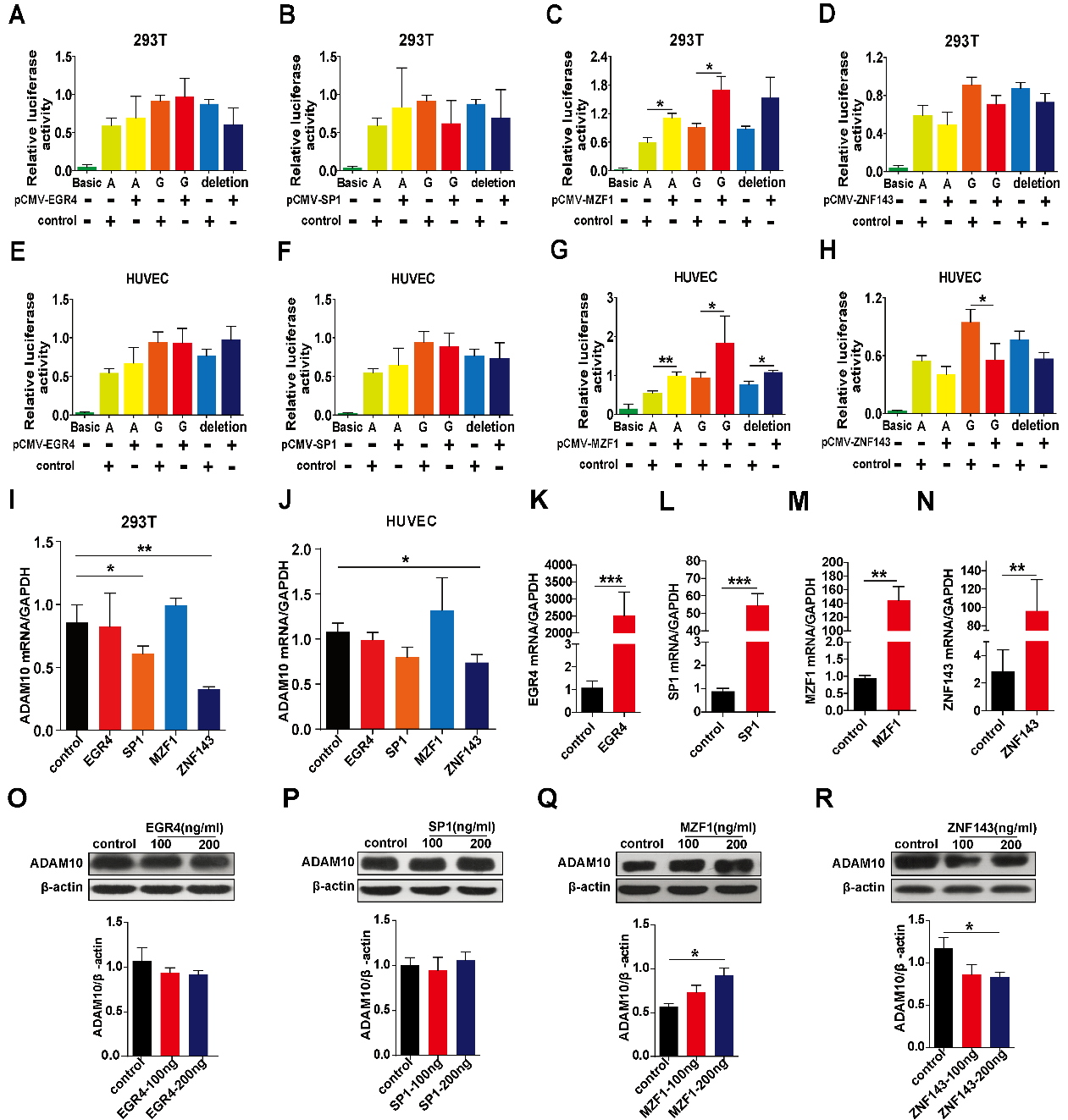 |
| --- |
| **Fig. S1. The screening of TFs and the Influence of TFs on ADAM10 expression in vitro.** Functional effects of the predicted TFs EGR4 (A and E), SP1 (B and F), MZF1 (C and G) and ZNF143 (D and H) on ADAM10 promoter activity in HEK-293T and HUVECs after cotransfection of each TF (1 ug/ml) with different haplotype carriers of the ADAM10 promoter (1 ug/ml). The effects of TFs (1 µg/ml) on ADAM10 mRNA expression in cultured HEK-293T (I) and HUVECs (J) were detected by qRT-PCR analysis. Expression levels of EGR4 (K), SP1 (L), MZF1 (M) and ZNF143 (N) were detected by qRT-PCR analysis after transfection with each plasmid (1 µg/ml) for 48 h in cultured HUVECs. The effects of EGR4 (O), SP1 (P), MZF1 (Q) and ZNF143 (R) on the expression of ADAM10 in cultured HUVECs for 72 h were detected by western blot analysis. The promoter activities were described as ratios of luciferase activities over renilla luciferase activities. The results of qRT-PCR and western blot analyses are presented as a fold of the control value. Data are presented as the mean ± SEM. At least three independent experiments were performed. *P < 0.05; **P < 0.01; ***P < 0.001. |
