# Supplementary material for: A Functional Polymorphism-Mediated Disruption of EGR1/ADAM10 Pathway Confers the Risk of Sepsis Progression
Source: mBio. 2019 Aug 6;10(4):e01663-19. doi: 10.1128/mBio.01663-19 (PMC6686044; doi:10.1128/mBio.01663-19)
Supplement: FIG S2 [file mBio.01663-19-sf002.docx]

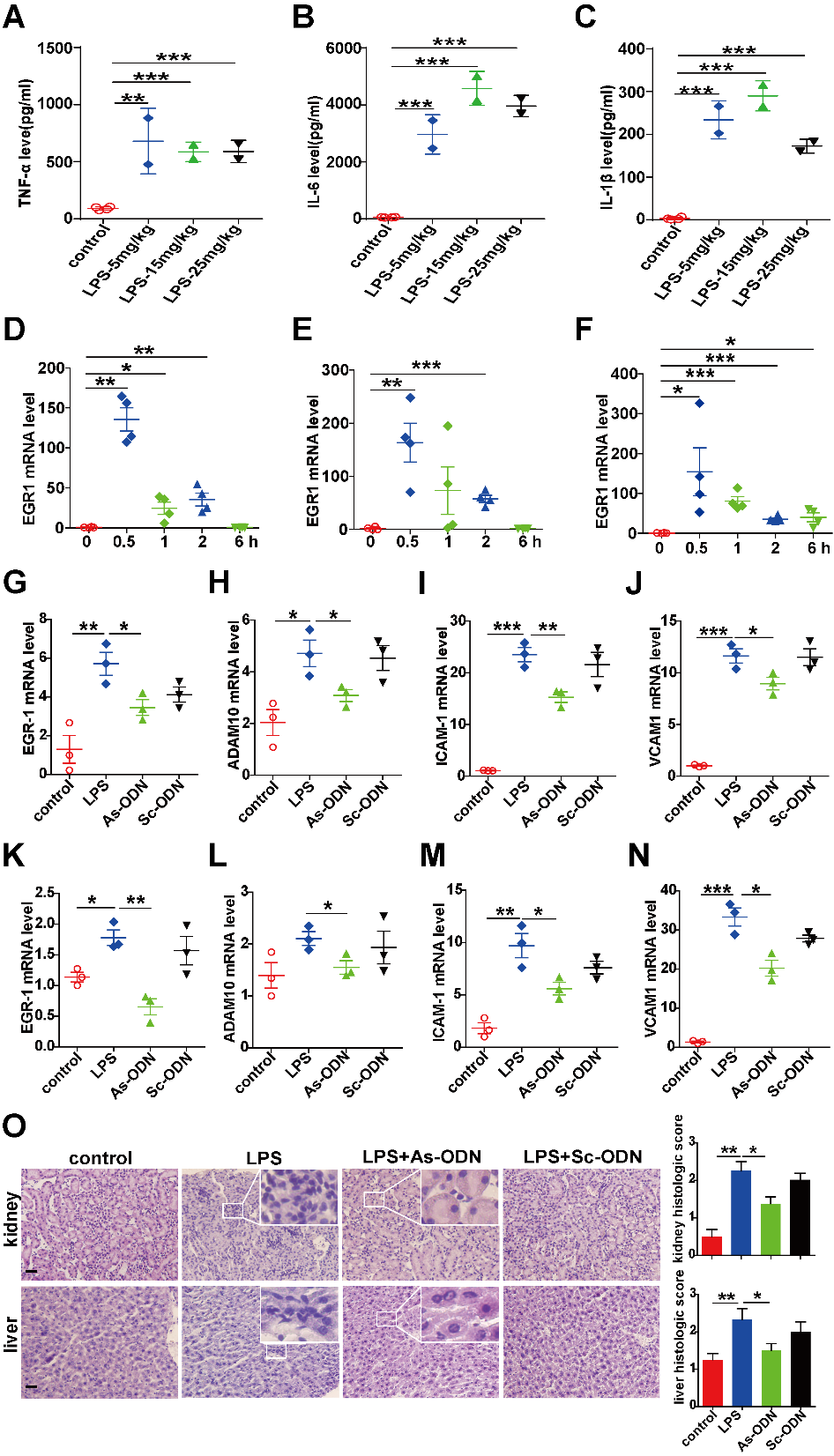


**Fig. S2. EGR1 As-ODN protects against the sepsis mouse model.** Blood samples were isolated 6 h after LPS injection, and serum was analyzed by ELISA for TNF-α (A), IL-6 (B) and IL-1β (C) release. Tissues were harvested in a time-dependent manner after LPS exposure to detect the EGR1 mRNA expression level (D-F). The mRNA levels of EGR1 (J, K), ADAM10 (H, L), ICAM-1 (I, M) and VCAM-1 (J, N) in kidney and liver tissues were determined by qRT-PCR, respectively. Histological changes in the kidney, lung and liver tissue sections from each group were stained with hematoxylin and eosin (H&E) (O). Scale bars, 50 μm for kidney and liver tissues. Images were taken at 200X magnification. Data are presented as the mean ± SEM (at least 3 animals per group). *P < 0.05; **P < 0.01; ***P < 0.001.
