# Supplementary material for: A Functional Polymorphism-Mediated Disruption of EGR1/ADAM10 Pathway Confers the Risk of Sepsis Progression
Source: mBio. 2019 Aug 6;10(4):e01663-19. doi: 10.1128/mBio.01663-19 (PMC6686044; doi:10.1128/mBio.01663-19)
Supplement: FIG S3 [file mBio.01663-19-sf003.docx]

| **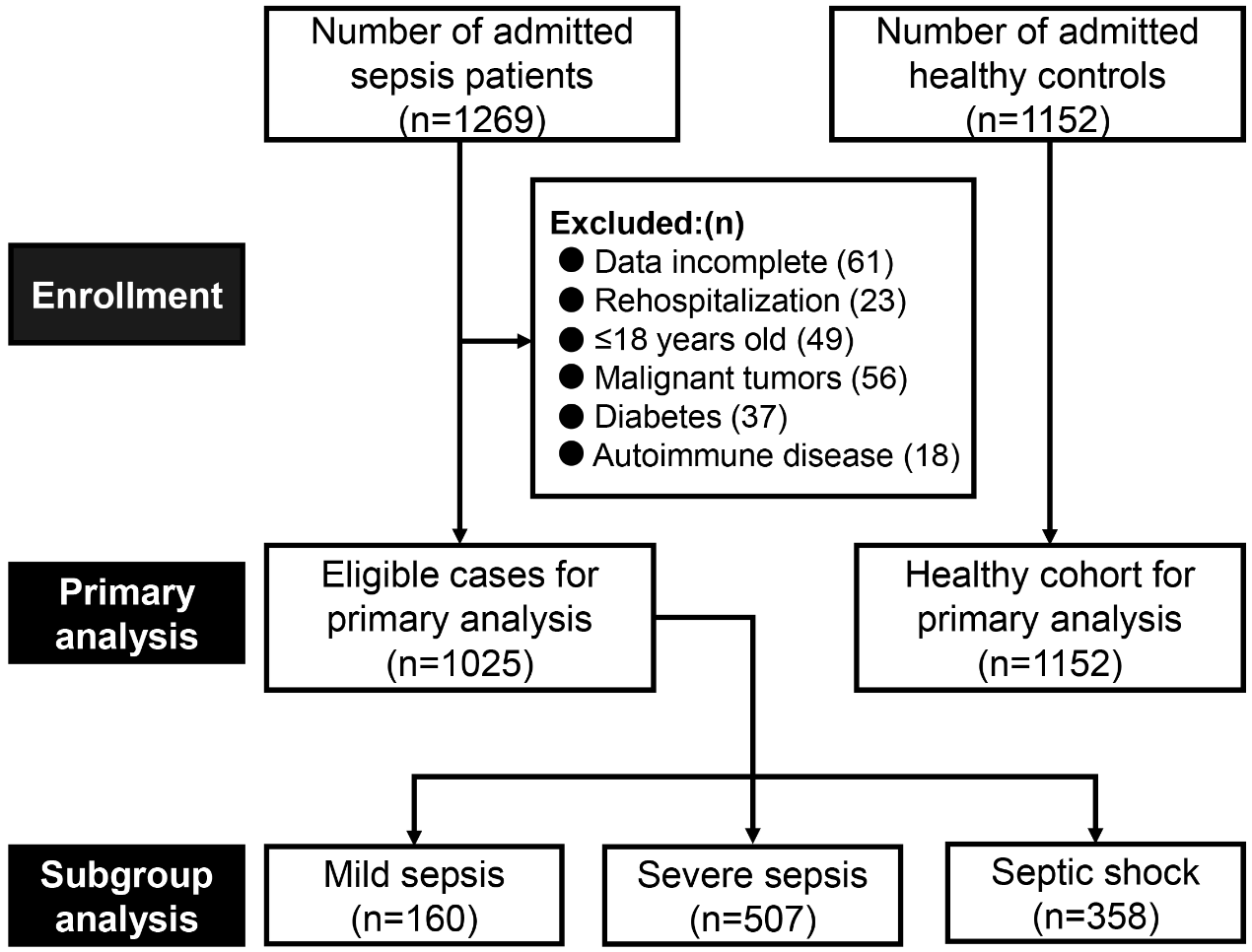** |
| --- |
| **Fig. S3. Flowchart of subject inclusion into clinical analysis cohort.** Main procedures of clinical studies include sample collection, primary analysis and subgroup analysis. The participants included 529 sepsis patients from the ICU and 610 healthy controls from the Affiliated Hospital of Guangdong Medical College in southern China (Zhanjiang, China), 111 sepsis patients and 139 healthy controls from the Center Hospital of Wuhan in central China (Wuhan, China) and 385 sepsis patients and 403 healthy controls from Harbin Medical University in northern China (Harbin, China). Among the 1269 patients diagnosed with sepsis, the following patients were excluded: 244 who did not meet the sepsis protocol, 61 in whom the data were incomplete, 23 duplicates, and those who were ineligible due to age, malignant tumors, diabetes and autoimmune disease, with 49, 56, 37 and 18 individuals, respectively. In total, 1025 sepsis patients and 1152 matched healthy controls were included in the primary analysis. A total of 1025 patients with confirmed sepsis and 1152 matched healthy controls were consecutively recruited from three representative regions of China. |
