# Supplementary material for: A Functional Polymorphism-Mediated Disruption of EGR1/ADAM10 Pathway Confers the Risk of Sepsis Progression
Source: mBio. 2019 Aug 6;10(4):e01663-19. doi: 10.1128/mBio.01663-19 (PMC6686044; doi:10.1128/mBio.01663-19)
Supplement: TABLE S4 [file mBio.01663-19-st004.docx]

| **Table S4. Primer sequences used for quantitative real-time PCR analysis**   \| **Species** \| **Genes** \| **Forward primer** \| **Reverse primer** \| \| \| --- \| --- \| --- \| --- \| --- \| \| **Human** \| *GAPDH* \| GCACCGTCAAGGCTGAGAAC \| \| TGGTGAAGACGCCAGTGGA \| \| *ADAM10* \| GCCCAGATATCCAGTCATGTTA \| \| GAAACTGATGTTACGGATTCCG \| \| *EGR1* \| GGTCAGTGGCCTAGTGAGC \| \| GTGCCGCTGAGTAAATGGGA \| \| *EGR4* \| TCCTCGTCAAGTCCACTGAAG \| \| CAGGAGTCGGCTAAGTCCC \| \| *SP1* \| TGGCAGCAGTACCAATGGC \| \| CCAGGTAGTCCTGTCAGAACTT \| \| *MZF1* \| TTCCGGTGCTTCCGCTATG \| \| CTCCTTGGAGCGTACCTCT \| \| *ZNF143* \| GTACAGGGGACAGTTTGCGTC \| \| TGGAGGTGTGGTGAATAAATGC \| \| *ICAM1* \| ATGCCCAGACATCTGTGTCC \| \| GGGGTCTCTATGCCCAACAA \| \| *VCAM1* \| GGGAAGATGGTCGTGATCCTT \| \| TCTGGGGTGGTCTCGATTTTA \| \| *VE-cad* \| TTGGAACCAGATGCACATTGAT \| \| TCTTGCGACTCACGCTTGAC \| \| **Mouse** \| *GAPDH* \| AATGGATTTGGACGCATTGGT \| \| TTTGCACTGGTACGTGTTGAT \| \| *ADAM10* \| GTGCCAAACGAGCAGTCTCA \| \| ATTCGTAGGTTGAACTGTCTTCC \| \| *Egr1* \| ACCCCTCTGTCTACTATTAAGGC \| \| TGGGACTGGTAGCTGGTATTG \| \| *Icam1* \| GTGATGCTCAGGTATCCATCCA \| \| CACAGTTCTCAAAGCACAGCG \| \| *Vcam1* \| TTGGGAGCCTCAACGGTACT \| \| GCAATCGTTTTGTATTCAGGGGA \| |
| --- | --- | --- | --- | --- | --- | --- | --- | --- | --- | --- | --- | --- | --- | --- | --- | --- | --- | --- | --- | --- | --- | --- | --- | --- | --- | --- | --- | --- | --- | --- | --- | --- | --- | --- | --- | --- | --- | --- | --- | --- | --- | --- | --- | --- | --- | --- | --- | --- | --- | --- | --- | --- | --- | --- | --- | --- | --- | --- | --- | --- | --- | --- | --- | --- | --- | --- | --- |
| Abbreviations: EGR1, Early Growth Response-1; EGR4, Early Growth Response-4; SP1, specificity protein 1; MZF1, myeloid zinc finger 1; ZNF143, zinc-finger protein 143; ICAM-1, intercellular adhesion molecule-1; VCAM-1, vascular cell adhesion molecule-1; VE-cad, VE-cadherin. |
